# Liposomal doxorubicin for active targeting: surface modification of the nanocarrier evaluated *in vitro* and *in vivo* — challenges and prospects

**DOI:** 10.18632/oncotarget.6191

**Published:** 2015-10-20

**Authors:** Judith Jakoby, Felix Beuschlein, Susanne Mentz, Constanze Hantel, Regine Süss

**Affiliations:** ^1^ Institute of Pharmaceutical Sciences, Department of Pharmaceutical Technology and Biopharmacy, Albert Ludwig University Freiburg, Freiburg, Germany; ^2^ Endocrine Research Unit, Medizinische Klinik und Poliklinik IV, Klinikum der Universität München, Munich, Germany

**Keywords:** Liposomal targeting, surface modification, Caelyx^®^, IGF1 receptor

## Abstract

Due to the inability of classical chemotherapeutic agents to exclusively target tumor cells, these treatments are associated with severe toxicity profiles. Thus, long-circulating liposomes have been developed in the past to enhance accumulation in tumor tissue by passive targeting. Accordingly, commercially available liposomal formulations of sterically stabilized liposomal doxorubicin (Caelyx^®^, Doxil^®^, Lipodox^®^) are associated with improved off-target profiles. However, these preparations are still not capable to selectively bind to target cells. Thus, in an attempt to further optimize existing treatment schemes immunoliposomes have been established to enable active targeting of tumor tissues. Recently, we have provided evidence for therapeutic efficacy of anti-IGF1R-targeted, surface modified doxorubicin loaded liposomes. Our approach involved a technique, which allows specific post-modifications of the liposomal surface by primed antibody-anchor conjugates thereby facilitating personalized approaches of commercially available liposomal drugs. In the current study, post-modification of sterically stabilized liposomal Dox was thoroughly investigated including the influence of different modification techniques (PIT, SPIT, SPIT60), lipid composition (SPC/Chol, HSPC/Chol), and buffers (HBS, SH). As earlier *in vivo* experiments did not take into account the presence of non-integrated ab-anchor conjugates this was included in the present study. Our experiments provide evidence that post-modification of commercially available liposomal preparations for active targeting is possible. Moreover, lyophilisation represents an applicable method to obtain a storable precursor of surface modifying antibody-anchor conjugates. Thus, these findings open up new approaches in patient individualized targeting of chemotherapeutic therapies.

## INTRODUCTION

Over the last two decades liposomes have been developed as potent carrier systems for several drugs including cytostatic agents [1]. There are several advantages in using liposomal encapsulated drug over the administration of the respective free drug. In case of Doxorubicin (Dox) for example encapsulation drastically alters pharmacokinetic properties resulting in extended half-life and in a reduced volume of distribution leading to decreased accumulation in healthy tissues and, ultimately diminishing side effects such as dose limiting cardiotoxicity [2–4]. Moreover, due to the enhanced permeability and retention (EPR) effect [5] Dox containing liposomes accumulate preferentially in tumor tissues leading to so called passive targeting effects [6, 7]. The prime example for such a liposomal product already clinically approved and on the market is sterically stabilized liposomal Dox (Caelyx^®^, Doxil^®^, Lipodox^®^) [8].

As shown in the past, the specificity and efficiency of the passive targeting can furthermore be increased by applying specific ligands to the liposomal surface resulting in an active targeting approach [9]. Depending on the ligand, such surface modification can result in endocytic uptake of the whole drug delivery system thereby enhancing intracellular drug delivery [10]. One promising therapeutic target that has been addressed by recent research is the IGF1 receptor (IGF1-R). Based on its strong expression in a wide range of human cancers and the important contribution of IGF1-R dependent effects on tumor biology [11, 12] different IGF-1-R targeting approaches have been developed with some promising results in preclinical and early clinical trials [12–16]. However, as it remains uncertain whether inhibition of IGF1-R signaling alone is sufficient to mediate sustained therapeutic effects, the combination with free cytotoxic agents has been initiated to complement effects of the targeted therapies [14, 17, 18].

Following this combinatory approach recently we have aimed at the development of anti IGF1-receptor antibody (1H7) coupled liposomal Dox to implement the therapeutic strategies as outlined above in one approach [19]. As IGF1-R dependent signaling has been shown to promote tumorigenesis and hypersecretion syndromes in neuroendocrine tumors of the gastroenteropancreatic system (GEP) [20–22], we investigated these IGF1-R-targeting immunoliposomes in a preclinical xenograft model with promising therapeutic potential for the treatment of GEP-NETs.

GEP-NETs are neoplasm with an estimated incidence of 5.25 per 100,000 population [23]. For patients with GEP-NET, surgery is considered as a curative treatment option. However, around 80% of newly diagnosed patients develop metastasis, requiring systemic treatment modalities [24]. The 5-year survival rates of patients with metastasized NETs is calculated to be 30% for NETs of pancreatic origin and 45% for NETs of ileal origin [25]. For rapid growing tumors chemotherapy represents a palliative therapeutic option. As the response rate to mono-therapy has been low, most chemotherapeutic regimens for neuroendocrine tumors rely on combination therapy [26–28]. However, while many reports have demonstrated modest sensitivity for pancreatic NET with response rates to chemotherapy ranging from 40 to 60%, metastatic carcinoid tumors with response rates of 20% are considered as poor responders to chemotherapy [23, 28]. Thus, additional treatment options for patients with GEP-NETs are urgently needed.

Therefore, the aim of our current study was further characterization of our recently developed anti-IGF1R targeting immunoliposomal therapy and furthermore the optimization of the post-modification approach. The possibility of post-modification of the commercially available drug Caelyx^®^ might provide a very promising possibility for patient-individual therapeutic targeting approaches in the future.

To improve the understanding for the influence of different parameters on the post-modification of liposomal drug delivery systems, liposomes in this study where either composed of SPC/Chol or HSPC/Chol/DSPE-mPEG (as in Caelyx^®^) and modified with either anti-GD2-ab (hu14.18, neuroblastoma model) or anti-IGF1R-ab (1H7, GEP-NET model). Two different anchor molecules were tested for integration of ab into liposomal membranes and three different techniques were applied for coupling (PIT, SPIT, SPIT60). Surprisingly, efficacy of post surface modification of commercially available Caelyx^®^ was strongly dependent on buffer composition, which was then thoroughly investigated.

## RESULTS

### Cellular association of 1H7 modified liposomes with BON cells

**Figure 1:**
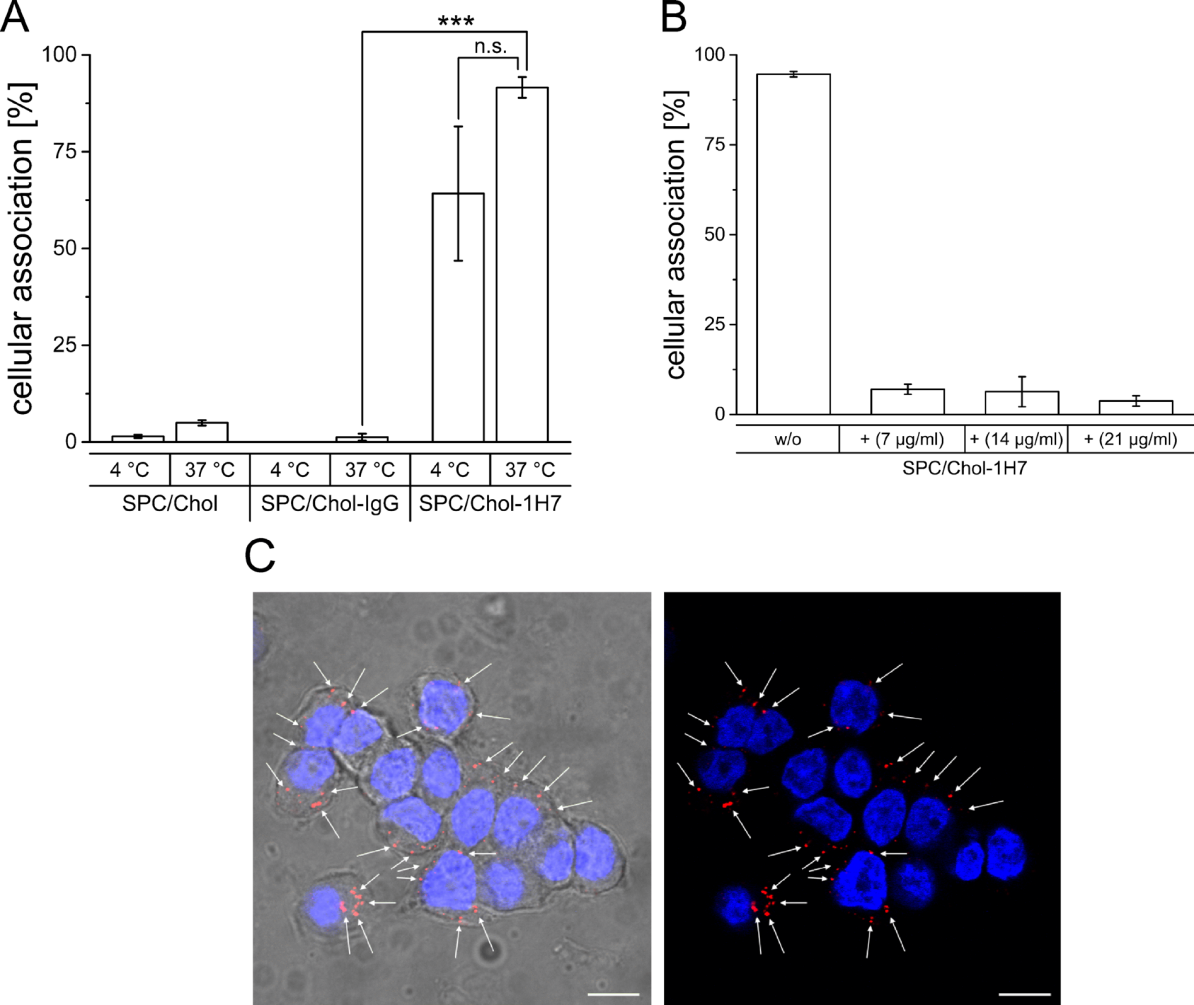
**(A)** Cellular association of Rh-PE labelled SPC/Chol liposomes with and without surface modification via SPIT using an unspecific IgG (SPC/Chol-IgG) or the specific 1H7 ab (SPC/Chol-1H7) at 37°C and 4°C(*n* = 3 ± SEM), **(B)** Cellular association of SPC/Chol-1H7 without (w/o) and with pre-incubation with free 1H7 ab in different concentrations (stated in the graph) (*n* = 3, ± SD), **(C)** Confocal micrographs of BON cells incubated with Rh-PE labeled SPC/Chol-1H7 liposomes (red) at 37°C and DAPI stained nuclei (blue)(63 x), Scale bar 10 μm.

In a first step, targeting ability, specificity and uptake route of anti IGF1-R directed liposomes using a cholesterol-based NHS-anchor was determined. Therefore, Rhodamine-PE labelled SPC/Chol liposomes were modified using a Chol-PEG-NHS anchor coupled with either the specific 1H7 antibody (SPC/Chol-1H7) or an unspecific IgG antibody (SPC/Chol-IgG). Cellular interaction was determined at 37°C and 4°C. While 37°C values represent passive and active cellular processes (like endocytosis), 4°C values only result from cellular attachment, as active metabolic processes do not occur at 4°C. As shown in Figure 1, control preparations (unmodified SPC/Chol and unspecific SPC/Chol-IgG) revealed no cellular association at 37°C (5.0 ± 0.7% and 1.3 ± 0.9%) as well as at 4°C (1.5 ± 0.4% and 0.0 ± 0.0%) indicating no binding or uptake of liposomes (Figure 1A).

In contrast, 1H7 modified liposomes revealed high cellular association at 4°C (64.2 ± 17.3%) and almost total association at 37°C (91.6 ± 2.7%) indicating that SPC/Chol-1H7 liposomes specifically bind to BON cells and that surface modification using an NHS activated Cholesterol-based anchor is applicable for our purposes. A decreased cellular association at 4°C suggests a metabolic active uptake process such as endocytosis at 37°C, which was confirmed by confocal microscopy (Figure 1C).

### Receptor mediated uptake

In order to clarify, whether the cellular uptake of SPC/Chol-1H7 is due to receptor mediated endocytosis, competition experiments with free 1H7 ab were performed to block the receptor. BON cells were pre-incubated with different concentrations of free 1H7 ab, followed by co-incubation with SPC/Chol-1H7 liposomes. The detected cellular association was drastically reduced after pre-treatment with free 1H7 (no pre-treatment: 94.6 ± 0.8%, 7 μg/ml: 7.0 ± 1.4%; 14 μg/ml: 6.3 ± 4.2%; 21 μg/ml: 3.7 ± 1.4%; Figure 1B) which provides evidence of a receptor mediated uptake.

### Adapting lipid and buffer composition to Caelyx^®^

For *in vivo* experiments it was intended to use liposomal Doxorubicin. As sterically stabilized liposomal Doxorubicin is already clinically approved and commercially available (Caelyx^®^), this product was investigated for its feasibility to be post modified for active targeting of tumor cells. Unlike the beforehand used SPC/Chol liposomes, which were dispersed in HBS, the HSPC-based Caelyx^®^ liposomes are dispersed in a histidine buffered isotone sucrose solution (SH). Thus, the influence of the lipid composition as well as the buffer system on surface modifications via PIT technique (DSPE-based anchor) or SPIT (Chol-based anchor) was investigated. As the 1H7 ab was not available in large quantities, another well established targeting model system was used: the neuroblastoma cell line Kelly (expressing the GD2 receptor) and the human anti GD2 antibody (hu14.18) [29].

**Figure 2:**
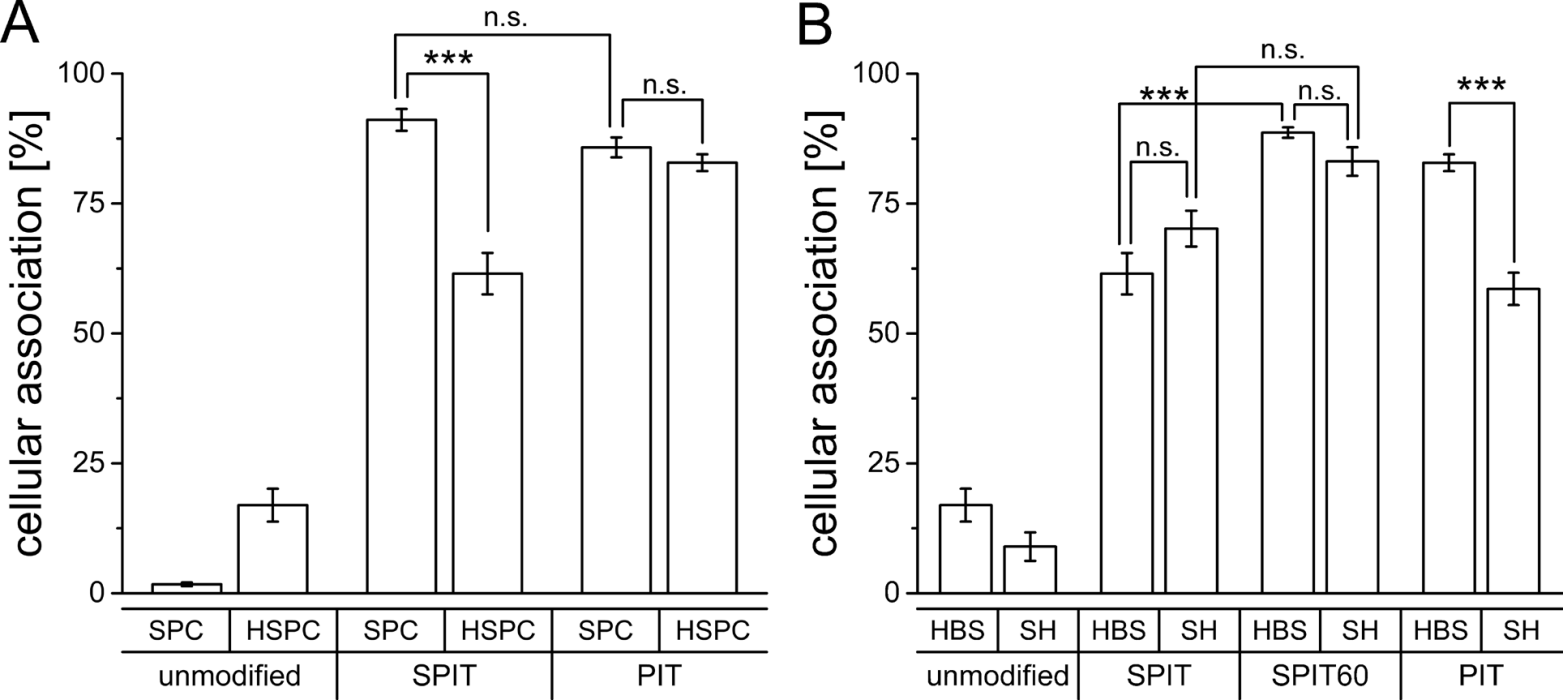
**(A)** Cellular association of Rh-PE labelled SPC/Chol (SPC) and HSPC/Chol/DSPE-mPEG (HSPC) liposomes dispersed in HBS and surface modified via SPIT or PIT utilizing an anti GD2 ab with Kelly cells at 37°C (*n* = 5 for SPC/Chol and *n* = 6 for HSPC/Chol/DSPE-mPEG ± SEM), **(B)** Cellular association of Rh-PE labelled HSPC/Chol/DSPE-mPEG liposomes dispersed in HBS or SH buffer, modified via PIT, SPIT or SPIT60 using an anti GD2 ab with Kelly cells at 37°C (*n* = 6 ± SEM).

Flow cytometry data revealed a comparable targeting efficiency of SPC/Chol liposomes for both anchor types (PIT 85.8 ± 1.9%; SPIT 91.1 ± 2.1%; Figure 2A) while liposomes composed of HSPC/Chol/DSPE-mPEG (Caelyx^®^ lipid composition) displayed significant differences in cellular association after surface modification via PIT and SPIT (PIT 82.9 ± 1.6%; SPIT 61.5 ± 4.0%; Figure 2A). Using PIT-modification, no significant differences in cellular association for both lipid compositions were determined (HSPC/Chol 82.9 ± 1.6%; SPC/Chol 85.8 ± 1.9%). This reduced targeting efficiency after SPIT might be due to a reduced insertion of the Chol-based anchor at 20°C compared to the insertion of the DSPE-based anchor at 60°C (as with PIT). As the phase transition temperature for HSPC is about 55°C [30, 31], a higher temperature should facilitate anchor insertion into the liposomal membrane. Thus, for further experiments the insertion temperature for the SPIT was also elevated to 60°C (SPIT60) to potentially improve anchor insertion.

In a next step the influence of the buffer system on anchor insertion was determined. For these experiments only the Caelyx^®^ lipid composition was used, as this was the composition intended to be used in the *in vivo* model. HSPC/Chol/DSPE-mPEG liposomes were surface modified via PIT, SPIT or SPIT60 and the cellular association was determined by flow cytometry. Cellular association for SPIT-modified liposomes buffered with either HBS or with SH was quite comparable (SPIT HBS 61.5 ± 3.99% vs SPIT SH 70.2 ± 3.4%; SPIT60 HBS 88.67 ± 1,01 vs SPIT60 SH 83.1 ± 2.7%; Figure 2B). Interestingly, HSPC/Chol/DSPE-mPEG liposomes dispersed in HBS displayed a significant superior targeting effect for SPIT modified liposomes when insertion temperature was increased to 60°C (SPIT 61.5 ± 3.9% vs SPIT60 88.7 ± 1.0%) indicating that insertion of anchor entity should be conducted above phase transition temperature of liposomes as well as anchor lipid. Surprisingly, cellular association was significantly reduced for PIT-modified liposomes using SH buffer instead of HBS buffer (SH 58.6 ± 3.1% vs HBS 82.87 ± 1.6%). Thus, these results demonstrated the SPIT method to be superior over PIT for surface modification of Caelyx^®^ liposomes.

### Influence of surface modification of Caelyx^®^ on size, PDI and EE

As mentioned before, the clinically approved drug Caelyx^®^ was intended to be used for *in vivo* experiments. Therefore, the influence of surface modification on the stability of this drug was investigated. The encapsulation efficiency of Dox in Caelyx^®^ liposomes is declared to be higher than 90%, leaving an amount of 10% of free drug. This amount of free drug should not be increased due to surface modification as the free drug is known to be responsible for higher side effects accompanied by less efficiency. Therefore, stability of encapsulation efficiency (EE) of Dox, liposomal size and polydispersity index (PDI) were investigated. Stability was analyzed after surface modification by PIT, SPIT andSPIT60 as well as after storage of the modified preparations at 4°C over a period of 6 weeks.

**Figure 3:**
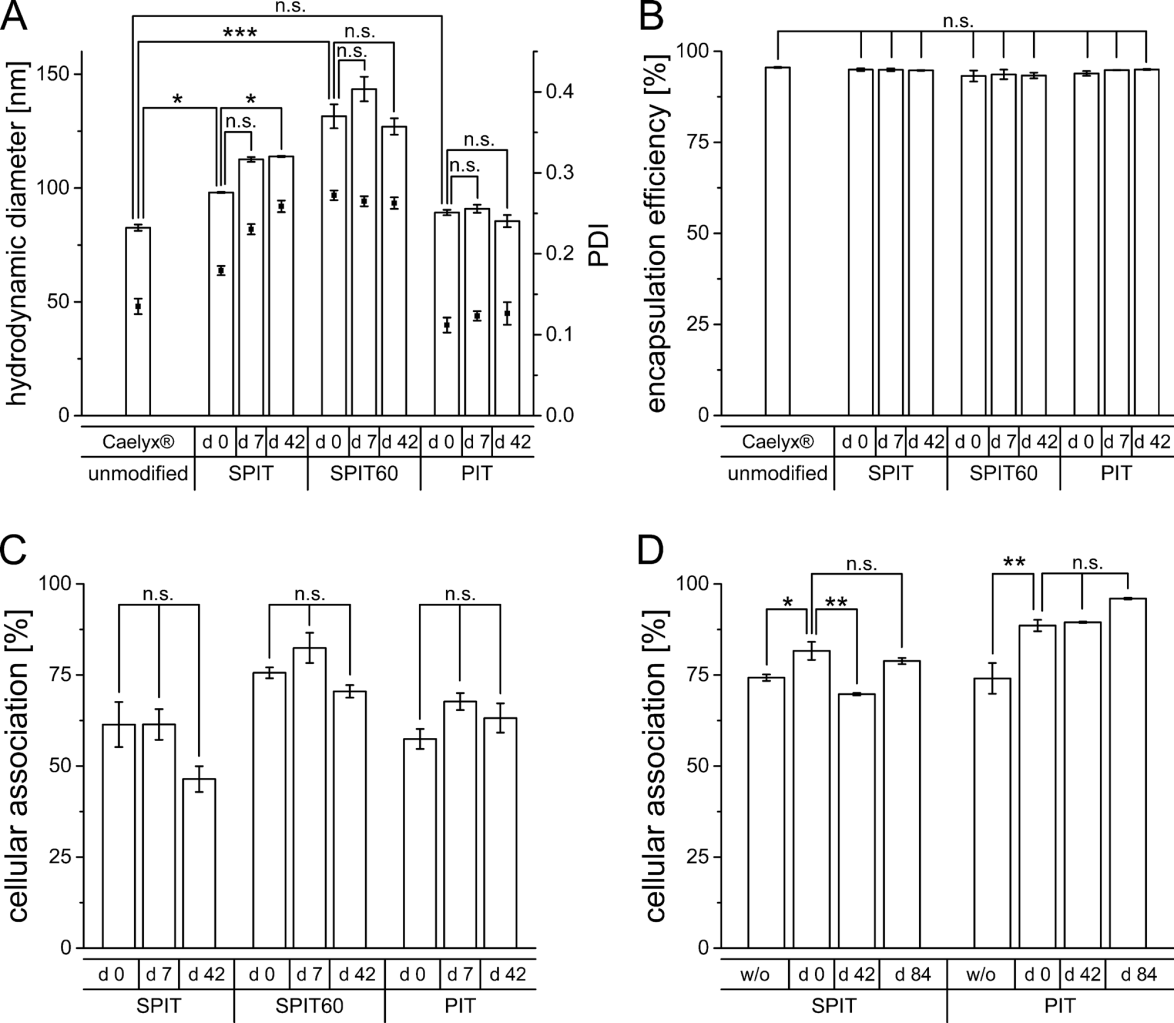
**(A)** Hydrodynamic diameter (primary y-axis) and PDI (secondary y-axis) of Caelyx^®^ determined by photon-correlation-spectroscopy before and after surface modification via SPIT, SPIT60 and PIT (d 0), as well as after 1 week (d 7) and 6 weeks (d 42) of storage at 4°C (*n* = 3 ± SEM), **(B)** Encapsulation efficiency of Dox in Caelyx^®^ before and after surface modification via SPIT, SPIT60 and PIT (d 0), as well as after 1 week (d 7) and 6 weeks (d 42) of storage at 4°C (*n* = 3 ±SEM), **(C)** Cellular association of Rh-PE labelled HSPC/Chol/DSPE-mPEG liposomes dispersed in SH buffer modified via SPIT, SPIT60 or PIT using an anti GD2 ab with Kelly cells at 37°C, immediately after production (d 0) and after 1 and 6 weeks of storage (d 7 and d 42), (*n* = 2 ± SEM), **(D)** Cellular association of Rh-PE labelled SPC/Chol liposomes dispersed in HBS modified via SPIT or PIT using an anti GD2 ab with Kelly cells at 37°C, without (w/o) and with lyophilisation of ab-anchor conjugate (d 0) and its storage over 6 (d 42) and 12 (d 84) weeks at 4°C (*n* = 3 ± SEM).

An increase in size was observed in all cases after surface modification, most pronounced for the SPIT60 preparation (Caelyx^®^ 82.6 ± 1.4 nm; PIT 89.3 ± 1.2 nm; SPIT 98,1 ± 0.2 nm; SPIT60 131.5 ± 5.3 nm; primary *y*-axis in Figure 3A). An initial increase in size after surface modification is reasonable due to ab size which is roughly about 10 nm in length [32]. After a storage of 6 weeks, sizes stayed stable for PIT and SPIT 60, whereas an increase was determined for SPIT preparations (PIT 85.5 ± 2.7 nm; SPIT 113.9 ± 0.3 nm; SPIT60 d 42: 127.0 ± 3.6 nm). Regarding PDI values, no significant changes were detectable for PIT, whereas an initial increase was observed for SPIT and SPIT60 preparations (Caelyx^®^ 0.135 ± 0.01, PIT 0.112 ± 0.009, SPIT 0.179 ± 0.006; SPIT60 0.272 ± 0.006; secondary *y*-axis in Figure 3A) The high PDI value for SPIT60 preparations indicates a rather inhomogeneous formulation that might not be suitable for *in vivo* use. The PDI for the PIT preparation (PIT d 42: 0.126 ± 0.014) as well as the SPIT60 preparation (SPIT60 d 42: 0.263 ± 0.007) stayed constant over 6 weeks. The SPIT preparation displayed a tolerable PDI at first, but debased over storage time (SPIT d 7: 0.230 ± 0.006; SPIT d 42: 0.259 ± 0.007), resulting in similar values as observed for the SPIT60 preparation right after surface modification. With regard to Dox encapsulation efficiency, neither anchor type nor insertion temperature had a negative impact. All three preparations still revealed good encapsulation efficiencies above 90% (Caelyx^®^ 95.6 ± 0.1%; PIT 93.9 ± 0.6%; SPIT 95.0 ± 0.4%; SPIT60 93.2 ± 1.5%) as well as storage stability over 6 weeks (Figure 3B).

### *In vitro* targeting efficiency after storage of surface modified liposomes

Targeting efficiency was evaluated after storage of the surface modified preparations. HSPC/Chol/DSPE-mPEG in SH buffer (displaying Caelyx^®^ model liposomes) were prepared and the surface was modified by PIT, SPIT, and SPIT60 with an anti-GD2 ab. Cellular association was evaluated after production (day 0) and after 1 and 6 weeks of storage at 4°C. For stored preparations size exclusion chromatography (SEC) of liposomes was performed prior to experiments to remove free ab-anchor conjugate, thus, the unpurified preparation was stored. This is in accordance with the preparations for *in vivo* studies, as for this preparation the non inserted ab-anchor conjugate is not removed as well. As it turned out, cellular association was not affected by storage for 7 days, whereas an, even though not significant, still reasonable reduction was observed for SPIT after storage of 6 weeks (PIT d 0: 57.4 ± 2.7%, d 7: 67.7 ± 2.3%; d 42: 63.2 ± 4.0%; SPIT d 0: 61.4 ± 6.2%, d 7: 61.4 ± 4.2%; d 42: 46.4 ± 3.5%; SPIT60 d 0: 75.6 ± 1.5%, d 7: 82.4 ± 4.1%; d 42: 70.5 ± 1.7%; Figure 3C). Thus, our experiments demonstrate the feasibility of all three methods to result in storable surface modified liposomal preparations for at least 7 days.

### Stability of ab-anchor conjugate

The possibility to prepare ab-containing liposomes on demand would be a very important step towards patient individualized therapy. Therefore, a protocol for the preparation of a storable, ready to use ab-anchor conjugate was developed. This ab-anchor conjugate could then be used for surface modification of any preformed liposomal drug. After ab-anchor reaction of anti GD2 ab with both anchor types (Chol- and DSPE-based) and the addition of histidine, the ab-anchor conjugate was freeze dried without the addition of any further substances and used for modification after rehydration. Control experiments were also performed with freshly prepared, not lyophilized ab-anchor conjugates (w/o lyo). Lyophilized ab-anchor conjugates were either rehydrated immediately after the freeze drying process (d 0) or after 6 and 12 weeks, respectively, inserted into the membrane of Rh-PE labelled SPC/Chol liposomes according to the SPIT or PIT protocol, and subsequently analyzed via flow cytometry. Only SPC/Chol liposomes were used for insertion of ab-anchor conjugates, as no attention to phase transition temperature had to be paid with this lipid composition.

For both ab-anchor conjugates (Chol-PEG-ab and DSPE-PEG-ab), lyophilisation did not impair the targeting efficiency of the ab. The cellular association for the preparations with lyophilized ab-anchor conjugate was even higher (SPIT w/o: 74.3 ± 0.9% and d 0: 81.6 ± 2.5%; PIT w/o: 74.1 ± 4.2% and d 0: 88.6 ± 1.6%; Figure 3D). Thus, lyophilisation of the ab-anchor conjugate is feasible. Cellular association of surface modified liposomes prepared from stored lyophilized ab-anchor conjugates revealed stable values over at least 12 weeks at 4°C (SPIT d 84: 78.8 ± 0.8%; PIT d 84: 96.0 ± 0.2%).

### Transfer of data to 1H7-BON model

**Figure 4:**
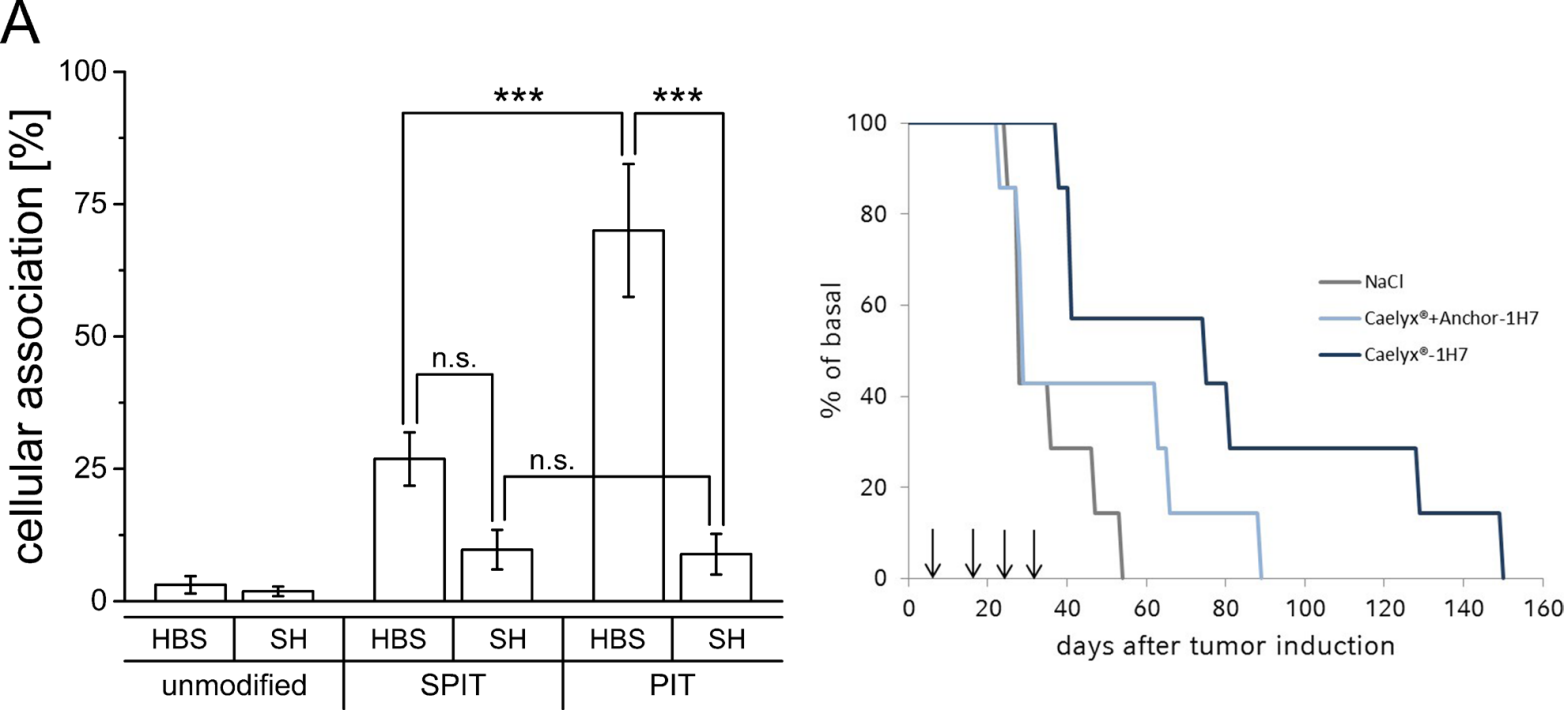
**(A)** Cellular association of Rh-PE labelled HSPC/Chol/DSPE-mPEG liposomes dispersed in HBS or SH buffer with BON cells at 37°C, surface modified via SPIT or PIT using the 1H7 ab, (n ≥ 4 ±SEM), **(B)** Overall survival for NaCl, Caelyx^®^ + anchor-1H7 and Caelyx^®^-1H7 treated BON tumor bearing mice after four treatment cycles on days 7, 17, 25 and 33 after tumor induction.

Results obtained from the GD2 model system revealed applicability of SPIT as well as PIT as suitable methods for surface modification of Caelyx^®^ liposomes regarding size and PDI as well as EE of Dox. The functionality of the surface modification itself was tested with empty liposomes composed of the same lipid mixture and dispersed in the same buffer system and resulted in high targeting efficiencies. Regarding storage stability, PIT was superior to SPIT, and SPIT60 was determined not to be suitable due to the high PDI indicating a polydisperse preparation. Thus, only SPIT and PIT were further evaluated for surface modification with the 1H7 ab in the GEP-NET system (BON cells). As it turned out, the targeting efficiency in this systems with this ab was in general much lower than achieved in the neuroblastoma model with the anti GD2 ab. Furthermore, stronger influences of both, lipid and buffer composition, were observed. Unexpectedly, for SH-buffered preparations almost no targeting effect was obtained (SPIT HBS: 26.9 ± 5.0% vs SH: 9.7 ± 3.8%; PIT HBS: 70.0 ± 12.5% vs SH: 8.9 ± 3.8%; Figure 4A).

### In vivo

As demonstrated, considerable effects were observed for the insertion of ab-anchor conjugates in liposomal preparations dispersed in SH buffer instead of HBS buffer. Less effective insertion consequently leads to a higher amount of free ab-anchor conjugate, which might be responsible for additional effects. As removal of free ab-anchor conjugate generally was not performed prior to the *in vivo* studies, additional control experiments with free ab-anchor were included in the study protocol. Therefore, and different to our previous studies [19] a control preparation was designed consisting of additional free anchor coupled 1H7-ab and not only free 1H7. This preparation was administered together with unmodified Caelyx^®^ (L + AK) in comparison to surface modified Caelyx^®^-1H7. Similar to earlier findings, a significant increase in overall survival in this new setting was still detectable for Caelyx^®^-1H7 compared to NaCl treated controls (*p* = 0.01) in the xenograft model for GEP-NETs, but significant differences in comparison to Caelyx^®^ + anchor-1H7 were diminished (*p* = 0.09). Moreover, repeated treatments did not lead to the expected additive increase in overall survival (Figure 4B).

### Buffer exchange for improvement of targeting efficiency

**Figure 5:**
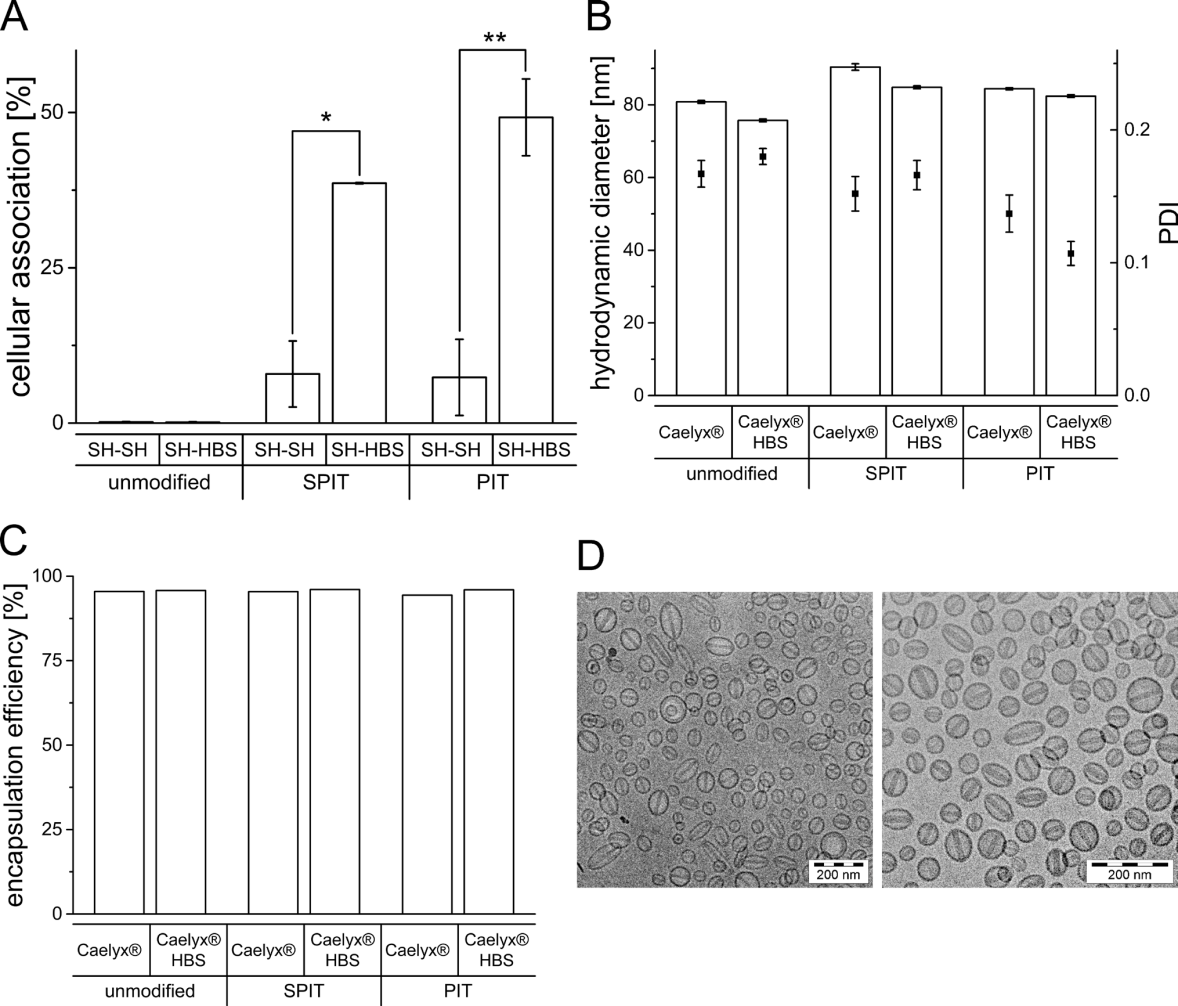
**(A)** Rh-PE labeled HSPC/Chol/DSPE-mPEG liposomes prepared in SH buffer and subsequent buffer exchange to SH and HBS, respectively. Cellular association with BON cells at 37°C, surface modified via SPIT or PIT utilizing the 1H7 ab (*n* = 2 ± SEM), **(B)** Hydrodynamic diameter and PDI determined by photon-correlation-spectroscopy of Caelyx^®^ and Caelyx^®^ HBS before and after surface modification via SPIT and PIT, **(C)** Encapsulation efficiency of Dox in Caelyx^®^ and Caelyx^®^ HBS before and after surface modification via SPIT and PIT, **(D)** cryo-TEM imaging of Caelyx^®^ before (left micrograph) and after buffer exchange to HBS (right micrograph).

As the presence of the SH buffer had an obvious negative effect on the surface modification of Caelyx^®^ it was further evaluated, whether exchanging the buffer would improve the targeting efficiency. Therefore, HSPC/Chol/DSPE-mPEG liposomes were prepared in SH and the external buffer was subsequently replaced by HBS using a vivaspin device. To also consider effects due to the vivaspin procedure itself, control liposomes were prepared in SH buffer, undergoing the vivaspin preparation with SH buffer again. After vivaspin procedure, surface modification via SPIT and PIT was performed and the cellular association of the preparations was determined via flow cytometry. As depicted in Figure 5A, an exchange of external SH to HBS (SH-HBS) resulted in significantly higher cellular associations for both methods compared to liposomes for which the external buffer was still SH (SH-SH) (SPIT SH-SH: 7.9 ± 5.3% vs SH-HBS: 38.6 ± 0.1; PIT SH-SH: 7.4 ± 6.1% vs SH-HBS: 49.2 ± 6.2).

### Buffer exchange with Caelyx^®^

It was furthermore evaluated, whether a buffer exchange is generally also feasible for Caelyx^®^ liposomes. The influence of changing the external buffer to HBS on liposomal size, PDI and Dox EE was determined with and without subsequent surface modification. Furthermore, cryo-TEM images were taken to eventually detect unwanted changes in morphology due to buffer exchange.

Regarding changes in size, no significant differences were detectable after buffer exchange and only minimal changes in PDI were observed (Caelyx^®^ HBS: 75.7 ± 0.4 nm; SPIT: 84.8 ± 0.4 nm; PIT 82.4 ± 0.4 nm; Figure 5B). Moreover, no differences in Dox EE were observed, indicating stability of liposomes despite buffer exchange from a sugar based to an ionic (NaCl) buffer (Caelyx^®^ HBS: 95.6%; SPIT: 96.0%; PIT: 96.0%; Figure 5C). Cryo-TEM images of Caelyx^®^ before and after buffer exchange depict uniform liposomes and show, that this procedure had no impact on the morphology of the liposomes. The visualization of the Dox crystals confirms the high encapsulation efficiency of Dox (Figure 5D).

## DISCUSSION

The aim of our current study was the detailed investigation and characterization of the influence of post-modifications for specific immunoliposomal targeting, using Caelyx^®^ liposomes and its lipid composition as basal formulation. Although sterically stabilized liposomes as Caelyx^®^ and pegylated immunoliposomes both reach tumor tissues by the same mechanism, passive targeting via the bloodstream, only immunoliposomes are capable to selectively bind to target cells [33, 34]. Moreover, despite the potential of enhanced therapeutic efficacy immunoliposomal preparations provide over unmodified liposomes the feasibility of personalized targeting approaches.

In recent years, the IGF1R has been suggested to be a promising target for different human cancers [12, 35]. Accordingly, IGF1R inhibitors were evaluated in preclinical and early clinical trials. However, although anti-tumoral activity of these agents has been demonstrated for several tumor entities there is a growing body of evidence that receptor blockage alone is not sufficient to induce a potent and sustained effect on tumor growth. Thus, combination with free cytotoxic agents has been used to complement effects of these targeted therapies [35].

In an attempt, to combine these different therapeutic strategies in a single formulation we developed recently anti-IGF1R-Dox loaded immunoliposomes and revealed enhanced targeting efficiencies against tumor cell lines of different human origin [19]. In our current study, we confirmed in a first step our previously obtained high targeting efficiencies of anti-IGF1R-ab SPIT-post-modified liposomes using a preclinical model for GEP-NETs (BON, Figure 1A). Moreover, as recent studies showed, that receptor down-regulation due to internalization is highly important for therapeutic efficacy of IGF1R-targeting approaches [35, 36] and as internalization of liposomes via a receptor mediated process furthermore has been shown to improve the nuclear delivery of encapsulated Dox to target cells leading to increased cytotoxic activity of the drug over non-targeted liposomes [34, 35], we investigated in a next step the cellular uptake route of our liposomal preparation. Our results provide evidence for a specific endocytic internalization of SPC/Chol-1H7 liposomes by BON cells by confocal microscopy *in vitro* (Figure 1C). In addition, cellular association was drastically reduced after pre-treatment with different free 1H7-ab concentrations indicating occupancy of IGF1-R sites on the cell surface resulting in a decrease of targeting efficacy (Figure 1B).

Apart from the uptake mechanism, many other factors are known to influence the therapeutic index and targeting efficiencies of liposomal encapsulated cytotoxic agents, such as lipid composition, liposome size, lipid dose, charge and forms of stabilization [37]. Consequently, we aimed at the investigation of the impact of specific lipids (SPC vs HSPC). Furthermore, as it turned out that even the buffer composition was highly relevant we also tested different buffer compositions (SH vs HBS) towards different applicable surface modifications (PIT vs SPIT vs SPIT60) and *vice versa* also the impact of these surface modifications on Caelyx^®^ liposomal size, PDI and encapsulation efficiency of Dox.

To the best of our knowledge, the detailed influence of surface modifications on Caelyx^®^ liposomes (SH-buffered HSPC/Chol/DSPE-mPEG) has not yet been investigated in detail and is an important prerequisite for a putative clinical translation of specifically targeted Caelyx^®^ -based immunoliposomes. As sterically stabilized liposomal Dox is under extensive investigation since many years showing anti-tumoral efficacy for a wide range of human cancers [38] and furthermore is already commercially available we intended to deploy this product for our purposes. Its main characteristics are the high Dox encapsulation efficiency (EE) of over 90%, the high stability and its prolonged plasma circulation time due to its unique lipid composition [39]. Thus, as the aim of any developed drug delivery system should be its applicability for a later use in humans, we are convinced that the optimization of an already proven Dox delivery system is advantageous.

Our experiments revealed a considerable influence of the lipid composition (SPC/Chol vs HSPC/Chol) on surface modification when deploying the SPIT method. Cellular association was significantly reduced for liposomes composed on the bases of Caelyx^®^, using HSPC/Chol compared to SPC/Chol liposomes (Figure 2A). When deploying the PIT method, no influence of the lipid composition was observed. As for the PIT technique the temperature is elevated during anchor insertion, we also used the SPIT at 60°C either (SPIT60), leading to comparable results as with SPIT with SPC/Chol liposomes (Figure 2B), while having a positive effect on HSPC/Chol/DSPE-mPEG liposomes. Our results indicate, thus, that the Chol-based (SPIT60) as well as the DSPE-based anchor [40] are generally feasible for surface modification of liposomes being composed of the same lipids as Caelyx^®^.

In recent years there was a rapid development of various targeted therapeutic approaches. Targets now include receptor tyrosine kinases (RTKs) (e.g. HER2, EGFR, MET), intracellular kinases (e.g. PI3K, MEK, AKT), transcription factors (e.g. STAT3), stem cell pathways (SHH/SMO, Notch), immunomodulators (e.g. CTLA4, PD1/PDL1, vaccines), and hormone receptors (e.g. estrogen, progesterone, androgen). However, despite advantages as high specificity or oral administration routes, they also bare specific challenges as stability or delivery problems and they furthermore often still require concomitant classic cytotoxic regimens for optimal therapeutic benefit [41]. Immunoliposomal preparations bear the potential to overcome these limitations for some of these targets. However, an important pre-requisite for such personalized approaches of an established liposomal agent is effective post-modification and ensured stability of the chemotherapeutic component, to not alter pharmacokinetic advantages of the established delivery system. In our experiments, we could demonstrate, that none of the surface modification methods had an impact on the encapsulation efficiency of Dox (Figure 3B). Size and PDI increased for both SPIT methods (Figure 3A). SPIT60 modification led to a substantial increase in the PDI immediately after preparation indicating a rather inhomogeneous preparation. This method was therefore judged as inappropriate for further use. For SPIT, increase in size and PDI was detected after a storage period of one week. Considering storage stability, only the PIT preparation turned out to be suitable for a storage over 6 weeks. Focusing on the stability of the surface modification and thereby the efficiency of targeting, all three methods proved to be stable (Figure 3C and 3D). Pursuing another option for obtaining a storable preparation, the ab-anchor conjugates were lyophilized and the stability was tested over a period of 12 weeks, revealing no significant changes in the their ability to enable cellular association after reconstitution and insertion into SPC/Chol liposomes (Figure 3D). Thus, lyophilisation represents an applicable method for obtaining a storable precursor of surface modified liposomes. This approach is an important prerequisite for the production of surface modified liposomes at the bedside. The shelf-life of Caelyx^®^ is not impaired as the product itself remains unchanged until surface modification. No further steps for the preparation of the ab-anchor conjugate has to be performed. Thus, only the storability of the ab-anchor conjugate have to be assessed and this conjugate can then be utilized for a wide range of targets using different ab types and preformed liposomal drugs [42]. Consequently, the insertion behavior of the ab-anchor conjugates into different liposomal species has to be assessed beforehand while storage stability of the surface modified product has not to be monitored as it will not be necessary to store the surface modified liposomal preparation.

Changing the buffer composition on the bases of Caelyx^®^ liposomes (from HBS to SH buffer) our experiments with HSPC/Chol/DSPE-mPEG liposomes unexpectedly indicated a remarkable impact on surface modification via PIT. Cellular association after PIT was significantly reduced while no influence for both SPIT methods was detectable (Figure 2B). First experiments had been carried out with an anti-GD2-ab neuroblastoma model system and were also performed with anti IGF1 R modified HSPC/Chol/DSPE-mPEG liposomes in a GEP-NET model. Interestingly, buffer impact was even higher in this system (Figure 4A). The same effect was also indirectly detectable in subsequent *in vivo* experiments (Figure 4B) using ab-anchor-conjugates as internal control instead of free 1H7 antibody. A significant increase in overall survival for Caelyx^®^-1H7 compared with NaCl treated controls was still detectable, but previously obtained significant differences in comparison to Caelyx^®^ + antibody were diminished [19].

Further attempt was focused on the question, whether an exchange of buffer might increase therapeutic efficacy. After replacement of the sucrose containing SH buffer by a NaCl containing (HBS), cellular association was indeed restored for both SPIT and PIT modified preparations (Figure 5A). These experiments confirmed that the reduced anchor insertion and therefore targeting ability of surface modified liposomes was impaired by the sucrose containing buffer of Caelyx^®^ liposomes and this is a very important finding for further optimization of immunoliposomal preparations. To evaluate, whether improved conditions would also increase efficiency of the targeting efficiency of Caelyx^®^, a buffer exchange was performed with this preparation. No significant differences in size, size distribution or encapsulation efficiency of Dox was detected after exchanging external buffer to HBS for unmodified and consecutively surface modified liposomes. Cryo-TEM images confirmed integrity of Caelyx^®^ liposomes (Figure 5B–5D). These findings demonstrate, that buffer exchange and therefore optimization of surface modification was also possible for Caelyx^®^.

In summary, we provide evidence that post-modification of commercially available liposomal preparations for active targeting is possible and these findings open up new approaches in patient individualized therapy. Further studies will focus on evidence of this approach in *in vivo* studies.

## MATERIALS AND METHODS

### Materials

Lipids, soy phosphatidylcholine (SPC), hydrogenated soy phosphatidylcholine (HSPC) and 1,2-distearoyl-*sn*-glycero-3-phosphoethanolamine-N-[methoxy(polyethylene glycol)-2000] (DSPE- mPEG) were kindly provided from Lipoid (Ludwigshafen, Germany). Lissamine™ Rhodamine B 1,2-dihexadecanoyl-*sn*-glycero-3-phosphoethanolamine (Rh-PE, Molecular Probes, Leiden, The Netherlands), cholesterol (Chol, Sigma Aldrich, Steinheim, Germany) and 1,2-distearoyl-*sn*-glycero-3-phosphoethanolamine-N-[N-succinimidylester (polyethylene-glycol)-2000] (DSPE-PEG-NHS, Nanocs, New York, USA) were furthermore used for preparation of liposomes as well as Chol-PEG-NHS which was synthesized in our lab. As sterically stabilized formulation of liposomal Doxorubicin the commercially available Caelyx^®^ (Doxil^®^) (Janssen-Cilag GmbH, Neuss, Germany) was used. The monoclonal antibody to CD221 (IGF1-receptor alpha chain, 1H7 clone) was purchased from Acris Antibodies (Hiddenhausen, Germany), the human anti-GD2 antibody (hu 14.18) was a generous gift from Prof. Dr. Rupert Handgretinger (University Children's Hospital Tübingen, Tübingen, Germany) Moreover, the unspecific IgG antibody from Sigma-Aldrich (Steinheim, Germany) was used.

### Liposome preparation

Liposomes for *in vitro* use were prepared by lipid film hydration method. They were either composed of SPC/Chol in a molar ratio of 7:3 or of HSPC/Chol/DSPE-mPEG in a molar ratio of 56:38:5. For analysis via flow cytometry 0.5 mol% of Rh-PE was added. Briefly, lipids were dissolved in Chloroform and solvent was removed in a rotary evaporator. The resulting lipid film was dried at minimal pressure for 2 h. Subsequently, the lipid film was hydrated by adding a buffer stated below resulting in a lipid concentration of 20 mM for SPC/Chol and 21.5 mM for HSPC/Chol/DSPE-mPEG liposomes. Two different buffers were used, HEPES buffered saline (HBS, 10 mM HEPES, 140 mM NaCl, pH 7.4) and a sucrose histidine buffer (SH, 10 mM histidine, 280 mM sucrose, pH 6.5). The dispersion obtained was homogenized by extruding 20 times through a polycarbonate membrane with 200 nm pores and 21 times with 80 nm pores using a hand-extrusion device (LiposoFast; Avestin, Ottawa, Canada). For HSPC/Chol/DSPE-mPEG liposomes all steps were performed in a water bath at 70°C and the buffer used was preheated. If Dox containing liposomes were needed, the commercially available pharmaceutical product Caelyx^®^ was used. HSPC/Chol/DSPE-mPEG liposomes as well as Caelyx^®^ already contain a pegylated lipid. Due to the more rigid bilayer and the presence of PEG chains on the surface the amount of ab-anchor conjugate was reduced to 1.25 mol% instead of 5% as in SPC/Chol liposomes.

### Surface modification of preformed liposomes

The surface of preformed liposomes was modified using either the sterol-based post-insertion technique [40, 43, 44] or the lipid-based post-insertion technique [40, 45, 46]. Either succinimide-activated Chol-PEG_2000_-NHS [40] or DSPE-PEG_2000_-NHS [40] was used as anchor. In brief, the ab solution (4–10 μg/μl) was pipetted into an anchor coated vial resulting in an ab/anchor ratio of 1 to 50 (mol/mol). The mixture was vortexed and bath sonicated for 30 s following incubation at 17°C under shaking (700 rpm). After 15 min the reaction was stopped by adding a 50 fold molar excess of histidine (in HBS, 20 g/l). After 10 min preformed liposomes were added in an anchor to total lipid ratio of 1 to 20 for SPC/Chol and 1:80 for HSPC/Chol/DSPE-mPEG liposomes, vortexed and depending on liposome composition and anchor type incubated as stated in Table 1.

**Table 1:** Parameters for anchor insertion depending on lipid composition and anchor type

Lipid composition	Anchor type	Insertion time	Insertion temperature (°C)	Method
SPC/Chol	Chol-PEG_2000_-NHS	30	17	SPIT
	DSPE-PEG_2000_-NHS	60	60	PIT
HSPC/Chol/DSPE-mPEG, Caelyx^®^	Chol-PEG_2000_-NHS	60	20	SPIT
		60	60	SPIT60
	DSPE-PEG_2000_-NHS	60	60	PIT

For cellular experiments, free ab-anchor conjugate was removed via size exclusion chromatography (Sepharose CL-4B), using the liposomal external buffer as eluent. Subsequently lipid concentration was determined by colorimetric phosphate assay according to Bartlett [47]. Total lipid concentration was corrected with regard to lipids not containing phosphate groups (e.g. Cholesterol). When modifiying Caelyx^®^ as liposomal dispersion for *in vivo* studies, all steps were performed aseptically under laminar flow and no separation step of free ab-anchor conjugate was performed.

### Stability of doxorubicin encapsulation after surface modification

To determine the stability of encapsulated Dox in Caelyx^®^ liposomes after surface modification, 2.5 μl of surface modified or unmodified Caelyx^®^ liposomal dispersion were added to 3 ml pre-heated HBS. The measurement was performed at 37°C under constantly stirring, all values were buffer background corrected. Fluorescence was measured at exc. 480 nm/em. 590 nm (slit 5 nm, LS 50 B, Perkin Elmer). As intraliposomal Dox fluorescence is quenched due to the high concentration, values only represent fluorescence of extraliposomal Dox (I_0_). Liposomes were destroyed by adding 10 μl of 10% (v/v) Triton X-100 and fluorescence of total Dox (I_100%_) was determined. Encapsulation efficiency (EE [%]) was calculated as EE [%] = 100 − (I_0_ / I_100%_ × 100). The storage stability at 4°C of surface modified Caelyx^®^ liposomes regarding EE of Dox was determined after 1 and 6 weeks, respectively.

### Cell culture

As *in vitro* model system the GD2 positive human neuroblastoma cell line (Kelly) was used. Kelly cells were obtained from the German Collection of Microorganisms and Cell Cultures (DSMZ, Braunschweig, Germany) and cultured in VLE RPMI 1640 medium (Biochrom, Berlin, Germany) supplemented with 10% (v/v) heat inactivated fetal calf serum (FCS, PAN-Biotech, Aidenbach, Germany). As IGF1-R expressing *in vitro* and *in vivo* GEP-NET model the human BON cell line was used. BON cells were obtained from the American Type Culture Collection (ATTC) and cultured in DMEM/Ham's F12 (1:1) supplemented with 10% (v/v) FCS. Cells were maintained at 37°C in an incubator with 5% CO_2_ and 95% of humidity. One day before the experiment, cells were seeded into 24-well culture plates at a density of 1.2 × 10^5^ (Kelly) or 8 × 10^4^ [48] cells/well, respectively. For confocal microscopy experiments, BON cells were seeded 24 h prior to the experiment on with 0.2% gelatin (in PBS) coated sterile cover glasses in a 24 well plate.

### Cellular association and uptake of Liposomes

One hour before incubation, medium was renewed. Cells were incubated for 2 hours with different liposomal preparations resulting in a lipid concentration of 150 μM. Subsequently, medium was removed, cells were washed with PBS and detached using Trypsin 0.5%/EDTA 0.25% (both from Biochrom, Berlin, Germany). For experiments using anti GD2 ab modified liposomes all supernatants were collected and transferred into tubes together with the harvested cells, as gangliosides play a role in cell adhesion [49]. When cells were treated with liposomes modified with an anti IGF1-R ab (1H7) only harvested cells were collected. Cells were spun down for 4 min at 1 200 rpm and 4°C, washed once with PBS and after a second centrifugation step resuspended in 200 μl PBS containing Ca^2+^ and Mg^2+^. Cells were kept at 4°C until analysis via flow cytometry (FACS Calibur^TM^, Becton Dickinson, Heidelberg, Germany). Results obtained were analyzed using CellQuest^TM^ Pro Software. To determine whether interaction of surface modified liposomes is due to an interaction with the IGF1-R, 30 min bevor adding the liposomal preparation free 1H7 ab was incubated with BON cells in different concentrations (7, 14, 21 μg/ml). For confocal microscopy, cells were washed twice after incubation and fixed with 4% (m/V) paraformaldehyde solution for 20 min at 37°C. Consecutively, cells were washed and nuclei were DAPI stained (150 nM, 5 min), washed again twice, mounted using MobiGLOW Mounting Medium and placed on microscope slides. Confocal Microscopy was performed using a Zeiss LSM 510 Meta microscope (Zeiss, Jena, Germany). Images were taking using a C-Apochromat 63x/1.2 W korr. Objective and analyzed via ZEN 2011 software.

### Lyophilisation of ab-anchor conjugate

Ab-anchor conjugate was prepared as stated above with the difference, that all steps were performed aseptically under laminar flow and only sterile solutions were used. After the reaction with histidine the obtained ab-anchor conjugate was divided into several vials and placed in the lyophilisator. Samples were frozen for 3 h at −45°C and ambient pressure followed by drying steps for 42 h at −20°C and subsequently for 6 h at 30°C under 0,05 mbar, respectively. Afterwards samples were stored at 4°C. Before usage they were rehydrated using purified water in an appropriate volume followed by insertion into preformed SPC/Chol liposomes. Suitability of lyophilisation for preservation of ab-anchor conjugates was evaluated via evaluation of targeting efficiency *in vitro*.

### Buffer exchange of preformed liposomes

External buffer of preformed liposomes was exchanged by using a vivaspin device (100 kDa cut off, PES membrane, Sartorius, Göttingen, Germany). Liposomes prepared in SH buffer were added into vivaspin tubes, spun down corresponding to manufacturer's protocol and washed three times with buffer stated as liposomal external buffer. Afterwards liposomes were removed and surface was modified as described.

### Therapeutic experiments

Female athymic NMRI nu/nu mice (6–8 weeks) were purchased from Harlan Winkelmann (Borchen, Germany) and housed under pathogen-free conditions. For tumor induction 15 × 10^6^ BON1 cells in a volume of 200 μl PBS were inoculated for tumor development subcutaneously into the neck of each mouse. BON cells were cultured as previously described [19]. For therapeutic experiments sterically stabilized liposomal doxorubicin (Caelyx^®^) was used as basal formulation. For immunoliposomal preparations the surface was modified via SPIT as stated above utilizing 1H7-antibody in a concentration of 10 μg/μl, resulting in Caelyx^®^-1H7. All steps were performed aseptically under laminar flow. As control treatment, first free ab-anchor conjugate and with a time lag unmodified Caelyx^®^ liposomes (designated as Caelyx^®^ + Chol-PEG-1H7) were applied. For ab-anchor conjugate preparation all steps were performed as described above unless liposome addition was omitted and dilution for administration was performed with sterile HBS resulting in a final volume of about 100–200 μl. Therapeutic treatments were applied intravenously for all groups (*n* = 7) at days 7, 17, 25 and 33 after BON tumor induction in dosages of 10 mg/kg body weight for sterically stabilized liposomal doxorubicin (Caelyx^®^) and 3.9 mg/kg for 1H7 antibody or the respective immunoliposomal preparation Caelyx^®^-1H7. To avoid methodological artefacts due to spontaneous aggregation, Caelyx^®^ + 1H7 were injected with a time delay of a few minutes into the same mouse. Antitumor effects were registered by measuring the longest tumor diameter. Mice were monitored daily and euthanized when the tumors reached a longest tumor diameter of 15 mm. All experiments were carried out following protocols approved by the Regierung von Oberbayern and in accordance with the German guidelines for animal studies.

### Statistical analyses

All results for the *in vitro* experiments are expressed as mean ± SEM unless stated otherwise. Statistical significance was determined using the one way ANOVA test (OriginPro software). Statistical significance is denoted as stars (*p < 0.05; **p < 0.01; ***p < 0.001) in the figures if not stated otherwise.

Statistical significance for the *in vivo* experiments was determined by survival curve analysis using Prizm software (Houston, TX). Statistical significance is denoted as stars (*p < 0.05; **p < 0.01; ***p < 0.001) in the figures if not stated otherwise.

## Abbreviations

1H7: anti IGF1-R ab
Ab: antibody
Chol: cholesterol
Dox: Doxorubicin
DSPE-mPEG: 1,2-distearoyl-sn-glycero-3-phosphoethanolamine-N-[methoxy(polyethylene
GD2: Disialoganglioside
GEP-NET: neuroendocrine tumors of the gastroenteropancreatic system; HBS, HEPES buffered saline
HSPC: hydrogenated soy phosphatidylcholine
PIT: post-insertion technique (in this context phospholipid-based)
Rh-PE: Rhodamine B 1,2-dihexadecanoyl-sn-glycero-3-phosphoethanolamine
SH: sucrose histidine buffer
SPC: soy phosphatidylcholine
SPIT: sterol-based post-insertion technique
